# Neuroinflammatory Loop in Schizophrenia, Is There a Relationship with Symptoms or Cognition Decline?

**DOI:** 10.3390/ijms26010310

**Published:** 2025-01-01

**Authors:** Claudio Carril Pardo, Karina Oyarce Merino, América Vera-Montecinos

**Affiliations:** 1 Laboratorio de Neuroinmunología, Facultad de Medicina y Ciencia, Universidad San Sebastián, Sede Tres Pascualas, Concepción 4080871, Chile; claudio.carril@uss.cl (C.C.P.);; 2 Departamento de Ciencias Biológicas y Químicas, Facultad De Medicina y Ciencia, Universidad San Sebastián, Sede Tres Pascualas Lientur 1457, Concepción 4080871, Chile

**Keywords:** schizophrenia, inflammation, cytokines, cognition, blood–brain barrier

## Abstract

Schizophrenia (SZ), a complex psychiatric disorder of neurodevelopment, is characterised by a range of symptoms, including hallucinations, delusions, social isolation and cognitive deterioration. One of the hypotheses that underlie SZ is related to inflammatory events which could be partly responsible for symptoms. However, it is unknown how inflammatory molecules can contribute to cognitive decline in SZ. This review summarises and exposes the possible contribution of the imbalance between pro-inflammatory and anti-inflammatory interleukins like IL-1beta, IL-4 and TNFalfa among others on cognitive impairment. We discuss how this inflammatory imbalance affects microglia and astrocytes inducing the disruption of the blood–brain barrier (BBB) in SZ, which could impact the prefrontal cortex or associative areas involved in executive functions such as planning and working tasks. We also highlight that inflammatory molecules generated by intestinal microbiota alterations, due to dysfunctional microbial colonisers or the use of some anti-psychotics, could impact the central nervous system. Finally, the question arises as to whether it is possible to modulate or correct the inflammatory imbalance that characterises SZ, and if an immunomodulatory strategy can be incorporated into conventional clinical treatments, either alone or in complement, to be applied in specific phases, such as prodromal or in the first-episode psychosis.

## 1. Introduction

### 1.1. Schizophrenia Prevalence

Psychiatric disorders, according to the Global Burden of Disease (GBD), constitute the main cause of the burden worldwide and the second cause of years of lived-with disability (YLDs). The last study carried out by GBD includes data from 1990 to 2019 showing an increase in mental disorders by over 48%. In this line, although schizophrenia (SZ) has a low incidence in the worldwide population, it was ranked in the top 25 mental disorders with more years lived with disability due to psychotic events [1], and a weighted average of 14.5 years of potential life lost [2] which substantially impacts the quality of life of patients and their families. For this reason, it is necessary to address and deepen investigation into the possible causes of this complex disorder to find therapeutic targets to improve the quality of life.

### 1.2. Aetiology of Schizophrenia

SZ is a complex psychiatric disorder in which neurodevelopment and genetic factors have a substantial role in its aetiology with a high heritability estimated at 79% [3]. The course of this disorder can present four phases: premorbid, prodromal, onset/deteriorative and chronic/residual [4,5]. The premorbid phase could start in gestation and continue from infancy to early adolescence with altered motor coordination and cognitive and social deficits [4]. The prodromal phase is present in adolescents and young adults; however, this phase does not have specific symptoms [6], although the presence of negative symptoms, positive-pre-psychotic and positive disorganisation have been reported [7]. Onset/deteriorative phase is characterised by the presence of negative and positive symptoms and cognitive dysfunction [8]; furthermore, this stage is where first-episode psychosis (FEP) occurs, which includes delusions and hallucinations [9]. The chronic/residual phase is characterised by psychosis, negative symptoms and significant neurocognitive deficits [10]. Although genetic factors have an important role in this disorder, several hypotheses have been proposed over the last decades to explain the pathophysiology of this complex neurodevelopment disorder. The first hypothesis proposed was (1) dopamine imbalance**,** formulated in the 1970s, due to the studies of the metabolism of dopamine and the discovery of antipsychotic drugs [11,12,13]. Later, (2) glutamatergic studies appeared in the 1990s showing that the N-methyl-D-aspartate (NMDA)-type glutamate receptors blocked by phencyclidine or ketamine-induced symptoms such as psychosis and cognitive disturbances, symptoms like SZ [14,15]. (3) The neurodevelopment hypothesis, proposed in 1988 by Weinberger, includes two critical periods of neurodevelopmental vulnerability, early life and adolescence, explained by the environmental double hit model, where a first hit, such as infection of the mother or stress during specific “time windows” in fetal development and a second hit during adolescence are required for the emergence of the disease [16,17,18]. (4) The neurodegenerative hypothesis is supported by studies that have shown that proteins involved in apoptosis in SZ such as anti-apoptotic protein Bcl-2 [19], overlap with other neurodegenerative diseases such as Alzheimer’s [19], Parkinson’s disease [20]. A recent study found fourteen autophagy-related genes as risk factors for SZ that could be related to mitigating neuronal damage, psychosis, or SZ-like behaviour. These genes are localised on chromosome 22q13, a region associated with SZ [21]. Although disseminated apoptotic events in the brain and cognitive deterioration in the later phases have been observed in SZ subjects, there has not been evidence of gliosis, a marker of neurodegeneration [22,23]. Nowadays, the neurodegenerative hypothesis is still not fully accepted.

In recent years, the neuroinflammation hypothesis has also been proposed as an event that could underlie this disorder, where inflammatory molecules could be involved in the brain–blood barrier (BBB) disruption and contribute to cognitive impairment in SZ subjects. The first evidence of a possible imbalance of the immune response in SZ subjects was presented three decades ago through the macrophage-T-lymphocyte theory [24] based on the production of specific interleukins such as IL-2, which could induce symptoms related to SZ precipitating the transition from prodromal to residual phase symptoms [25].

A point to highlight is the effect of proinflammatory cytokines on neurotransmitter synthesis or neuroactive metabolites. It has been reported in a recent study carried out by Zhang et al., [26] in drug-naive first-episode (FEP) SZ subjects, that high levels of IL-1β, IL-6, TNF-α, IL-17, IL-4, and IFN-γ induce high levels of enzyme indoleamine 2,3 dioxygenase (IDO), which degrade tryptophan (important for survival and protein synthesis) through the kynurenine pathway, producing kynurenic acid, a precursor and antagonist of N-methyl-d-aspartate (NMDA) receptors. Increased levels of IDO are positively correlated with negative symptoms in SZ. In this context, altered NMDA signalling underlies SZ and thus a constant inflammatory environment can exacerbate negative symptoms in SZ.

Moreover, the SARS-CoV-2 infection was a recent event that showed a link between immune responses and SZ symptomatology, probably due to an imbalance in the immune response and an increase in pro-inflammatory molecules. SZ subjects who contracted SARS-CoV-2 showed worsening negative and positive symptoms [27]. This could be explained by the diathesis-stress hypothesis, where the impact of a nervous system infection induces microglia activation, which in turn promotes neuroinflammation contributing to the loss of cortical grey matter and disinhibition in subcortical dopamine, contributing to the negative and positive symptoms, respectively, [28] in SZ. Furthermore, a study proposed that immune response dysfunction and inflammatory storm in healthy subjects exposed to SARS-CoV-2 could be a risk factor for developing SZ [29]; however, more studies are necessary to determine if this is true or whether prenatal infection with SARS-CoV-2 is another risk factor for developing SZ in adolescence or adulthood.

The question arises whether subjects with SZ exacerbated immune response inducing an uncontrolled increase in pro-inflammatory molecules, which induces the BBB disruption, and leads to neuronal damage and cognitive deterioration in SZ. According to the available evidence, this review shows an exhaustive description of anti-inflammatory and pro-inflammatory molecules reported in SZ and their relationship with cognitive damage due to possible blood–brain barrier disruption.

## 2. Neuroinflammatory Imbalance in Schizophrenia

### 2.1. Dysregulation of Proinflammatory Molecules

The evidence on immune dysfunction and inflammation in SZ is supported by studies where high levels of inflammatory molecules during pregnancy, due to maternal infection, could affect fetal development increasing the risk of SZ [30]. Studies on cerebrospinal fluid (CSF) also have shown high levels of pro-inflammatory molecules [31] and vascular endothelial growth factor, this protein increases BBB permeability in SZ [32]. Further, the Human Genome-wide Association study (GWAS), has identified genetic variants in SZ involved in the immune response such as the major histocompatibility complex (MHC) locus and Toll-like receptors (TLRs) [33].

In the context of inflammatory molecules, cytokines are the main actors. Studies have demonstrated that the imbalance of cytokines expression could be a central axis in SZ, cytokines mediate the communication between immune cells and neuronal cells. The cytokines are comprised of interleukins (IL), interferons (INF), chemokines, tumor necrosis factors (TNF), and transformation growth factors (TGF) [34].

#### 2.1.1. TNF-α

Studies in this area have shown that TNF-α, released by macrophages during acute inflammation [35], increases in SZ and the FEP; moreover, the upregulation of TNF-α is correlated with negative symptoms [36,37]. A meta-analysis study found a possible polymorphism in the gene *TNF-α*, which could generate susceptibility to SZ [38].

#### 2.1.2. IL-2

Some IL play a role in the proliferation and maturation of immune cells, increasing their activity in inflammatory processes [39]. In SZ, numerous pro-inflammatory ILs have been related to this disorder, among them, ow levels of IL-2, which is important for the function and survival of regulatory T cells (Treg), cells that regulate the innate and adaptative immune response. High levels of IL-2 could increase the immune response and promote the release of pro-inflammatory cytokines. Moreover, genetic studies have reported that polymorphism del gen IL-2 could be related to an increased risk of developing SZ [40].

In SZ, IL-2 has been related to negative symptoms and the progression of the disorder [41,42]. The mechanism of how IL-2 influences symptoms in SZ could be due to the effect of IL-2 on the locus coeruleus (LC) a region that participates in the executive task and has a vital function in negative and positive symptoms in SZ [43]. In this context, LC has IL-2 receptors and animal studies have demonstrated that microdoses of IL-2 induce aggressive behaviour in rats, which decreases with the administration of antipsychotic drugs [24]. However, the molecular pathway through which IL-2 influences positive and negative symptoms is unknown.

#### 2.1.3. IL-1β

IL-1β is a pro-inflammatory molecule involved in the stress response in the central nervous system, its exogen administration can impair memory, functions, and long-term potentiation (LTP) induction [44], damaging learning. In this context, a study carried out on FEP showed that high levels of IL-1β in serum correlated to the general psychopathological score on the Positive and Negative Syndrome scale (PANSS) for SZ [45] and with ultra-high risk (UHR) psychosis subjects [46]. Further, IL-1β administration to rats induces an increase in dopamine levels in the nucleus accumbens (NA) and decreases them in the prefrontal cortex (PFC) [47]. In SZ subjects, dopaminergic hyperactivity in the NA is related to auditory and verbal hallucinations [48], while hypoactivity in the PFC is related to cognitive dysfunction [49,50]. Therefore, some symptoms related to positive and deteriorated cognition seen in SZ could be a response to the increase in IL-1β and its influence on neurotransmitter dysregulation. The polymorphism of IL-1β on SZ has also been studied, and the results have determined some *IL-1β* polymorphisms such as rs1143633; however, this polymorphism is only found in SZ in Japanese women [51], and the *IL-1β-511* polymorphism is present in Caucasian Finnish SZ subjects [52].

#### 2.1.4. IL-5

IL-5 is associated with a pro-inflammatory immune response, as it activates eosinophils [53]. Higher IL-5 levels have been detected in SZ subjects, with a significant increase in patients from 18 years old who received antipsychotic treatment. There was even a significant increase in IL-5 in serum, rather than in CSF, a situation that is repeated for other cytokines [54]. Despite medication, SZ patients exhibit increased levels of IL-5 and other cytokines, even anti-inflammatory cytokines, which may be part of a counter-regulatory response, showing a degree of imbalance in the immune response of SZ subjects.

#### 2.1.5. IL-6

On the one hand, IL-6 is produced by macrophages and glial cells. IL-6 is a pleiotropic cytokine involved in development and neuronal differentiation but may also induce brain damage [55]. A meta-analysis found that IL-6 levels are increased in serum, plasma, and CSF in samples from SZ subjects [56]. On the other hand, in SZ subjects without psychotic treatment, IL-6 was found to be upregulated in serum in comparison with healthy controls; however, in SZ subjects with antipsychotic prescriptions, the IL-6 levels were decreased by the effect of antipsychotic treatment [57]. A functional study of SZ subjects suggests that increases in peripheral IL-6 levels could be related to poorer facial emotion recognition (Theory of Mind) due to dysfunctional neuronal connectivity in areas involved in facial emotion recognition such as the insula [58,59]. However, different factors such as smoking habits, sedentary lifestyle, sex and infections could alter IL-6 levels in SZ subjects. Age is also an important factor that increases IL-6 levels, which is referred to as “cytokine of gerontologists” [60]. Therefore, more studies are required to elucidate whether IL-6 plays a role in SZ, for example, through emotion recognition dysfunction related to negative symptoms.

#### 2.1.6. IL-8

IL-8 is synthesised by neurons, microglia, astrocytes and endothelial cells [61]. IL-8 is a chemokine that participates in the brain as a chemoattractant for T cells in response to injury [62]. IL-8 has been related to several psychiatric disorders such as depression, bipolar disorder, cognitive disorders, autism spectrum disorder and SZ. High levels of IL-8 have been found in drug-free SZ subjects, while in SZ subjects receiving antipsychotic treatment, the IL-8 level was decreased [63]. The effects of high IL-8 levels in the CNS have been associated with decreased grey matter volumes [64], which could be associated with cognitive deterioration in subjects SZ.

#### 2.1.7. IL-16

Neuronal IL-16, expressed in the cerebellum and hippocampus and produced by a subpopulation of microglial cells has been reported to act as a chemoattractant for CD4+ lymphocytes to the BBB during brain injuries [65]. IL-16 may have a pro-inflammatory effect [66] and an anti-inflammatory effect [67]. An increase in IL-16 has been related to psychiatric disorders as the first onset of major depression and suicidal behaviours [68]. Studies have reported high levels of IL-16 in SZ subjects following psychotic events [69] and a positive correlation with negative symptoms [45]. Recently, the possible correlation of serum inflammatory factors with symptomatology in different stages of SZ has been compared, including first-episode SZ (FES) subjects, recurrent SZ subjects and relapse-episode SZ. Among these factors, IL-1β and IL-16 were highlighted, with IL-16 levels significantly higher in both the FES and relapse-episode groups compared to healthy controls. However, there was no significant difference between the FES and relapse-episode subjects. In the relapse-episode group, serum IL-16 levels were positively correlated with the negative symptoms of the PANSS scale and negatively correlated with the composite score (COM), which could be a clear inflammatory response. This suggests that the level of IL-16 could be an important signal to identify the risk or understand the development of SZ throughout different stages of the pathology. However, the molecular mechanism by which IL-16 exerts its action on the symptomatology of SZ has not been studied.

### 2.2. Dysregulation of Anti-Inflammatory Molecules

As previously mentioned, in the effort to elucidate the aetiology of SZ, there is straightforward evidence that an imbalance in the immune response, both peripherally and in the CNS, is highly relevant. We have already summarized the evidence considering pro-inflammatory molecules, but the question of what is happening with the anti-inflammatory molecules is yet to be addressed. These molecules have been discussed to a lesser extent than the pro-inflammatory ones, but their incidence in SZ is important to highlight and we will discuss some of them below.

IL-10 and transforming growth factor (TGF-β) are two of the most classical anti-inflammatory cytokines, crucial for maintaining immune system homeostasis. Under inflammatory contexts, these cytokines can be found to be decreased; however, the opposite can also be observed as it has been proposed that activation of anti-inflammatory pathways might be a homeostatic response to counteract inflammation [70].

#### 2.2.1. TGF-β

Most of the studies on TGF-β show that SZ subjects have high levels in plasma [71,72], even those patients classified as acutely relapsed and FEP [70]. Interestingly, in one study, high levels TGF-β in serum were found to be inversely correlated with lateral occipital cortical thickness, visual learning and memory scores and were also negatively associated with the MATRICS Consensus Cognitive Battery (MCCB) scale for visual learning and memory and social cognition in SZ subjects [73]. Moreover, high levels of TGF-β1 mRNA and protein in SZ subjects are associated with structural changes in the brain and deficits in cognitive functions, especially in vision-related areas, which is quite striking, especially coming from a neuroprotective cytokine. Other recent studies, in the context of ageing and memory, show that an increase in TGF-β1 is associated with improved structural conservation of the hippocampus, especially of the dentate gyrus, in older adults, which is related to better episodic memory performance [74]. Furthermore, it has also been studied whether polymorphisms in the *TGFB1* gene can serve as a molecular marker for susceptibility to SZ, as it was determined that the polymorphisms +869T > C would be implicated as a risk factor for developing SZ, by increasing the risk more than two-fold if the patient is a carrier of the T allele [75]. In addition, a significant decrease in TGF-β levels was observed after antipsychotic treatment, indicating that it could be used as a psychotic state marker [70]. This observation correlates with the finding of one study that reported lower levels of TGF-β in blood from patients with treatment-resistant SZ [76]. However, the action of antipsychotics and its anti-inflammatory effect on TGF-β levels should be studied.

#### 2.2.2. IL-10

In drug-naive first-episode SZ subjects, a significant decrease in IL-10 was observed, and there was an inverse correlation with negative symptoms from the PANSS scale [77], which could suggest a possible relation between IL-10 decrease and cognitive impairment. This situation has already been reported in patients with FEP [78]. However, contradictory observations have also been reported, because IL-10 increases have been detected in serum from SZ subjects, where these increases have been studied in conjunction with white matter (WM) integrity. Using serum samples and diffusion tensor imaging (DTI) from SZ subjects, it was observed that patients with increased IL-10 showed evidence of WM compromise, which would result in the disruption of fibre tracts or demyelination in some areas, involving the right posterior thalamic radiation and the left inferior frontal-occipital fasciculus [79]. It is known that the prefrontal cortex sends and receives nerve fibres to different areas, such as parietal, temporal, and occipital regions, areas that are also affected in SZ [80,81,82]. These observations support previous studies reporting a reduced magnetisation transfer ratio, the disruption of WM structure by abnormalities in the axonal structure and decreased myelin content in SZ subjects [83]. In fact, the different areas affected by WM detriment are also affected in SZ. Alterations in the temporal lobe generate problems in hearing and language processing [82], which could be related to positive symptoms, while alterations in the parietal lobe also led to cognitive disorders, visuospatial attention, and praxis abilities, among others [84]. 

Genotyping of different polymorphisms as a marker of susceptibility to SZ for IL-10 has been also performed and studies have found a possible correlation between peripheral *IL-10* levels and the -592 A/C polymorphism, where the -592 A/C allele was significantly increased in SZ subjects. It was determined that this allele would be related to a decrease in IL-10 in serum and attentional deficit, increasing the probability of FES. Also, these low levels directly correlated with Repeatable Battery for the Assessment of Neuropsychological Status (RBANS) cognitive tests, so this polymorphism could affect neurocognitive functions in SZ subjects [85]. Thus, low IL-10 levels could translate into low anti-inflammatory activity in SZ subjects, with other health implications. However, more studies are necessary because the existence of the -592 A/C polymorphism will depend on the population or ethnic group studied [86]. This is because the relevance of the same polymorphism has been previously reported in patients from Turkic populations [87], in addition to the potential of 26 other polymorphisms in subjects of African American, Hispanic American and European American ancestry [86].

We found one animal study with a rodent model of schizophrenia where TGF-β and IL-10 were evaluated but in the hippocampus. In this study, maternal immune activation (MIA) was used by challenging the pregnant dams with LPS or polyinosinic-polycytidylic acid (poly I: C), and the offspring developed neurobiological and behavioural alterations consistent with SZ [88]. The authors observed an increased expression of TGF-β and IL-10 at the RNA level but not at protein levels.

#### 2.2.3. IL-4

We also can highlight IL-4, a cytokine participating in the type 2 immune response [89], which is known to have an anti-inflammatory effect [90]. Slight increases in IL-4 and IL-10 concentrations have been observed in blood samples of patients with FEP, showing correlation with negative symptoms according to the PANSS scale [91]. Also, in patients suffering a psychotic break in acute exacerbations of SZ, the concentration of IL-4 is significantly increased; however, no correlation was found with either positive or negative symptoms according to the PANSS scale [92]. In SZ patients in relapse, upon receiving antipsychotic treatment in the form of a single antipsychotic or a combination of two antipsychotics such as haloperidol and chlorpromazine, IL-4 levels decreased significantly [93]. Research related to allelic variations, in the form of polymorphisms, has also been developed to determine whether this interleukin could be a molecular marker; however, the analysis of the serum of patients with bipolar disorder and SZ reveals that polymorphisms in the promoter gene of *IL-4* or its receptor are not associated with SZ in populations from different countries and ethnic origins [40,94,95,96]. Therefore, *IL-4*-associated polymorphisms would not be a good indicator of susceptibility or risk factor to SZ.

#### 2.2.4. IL-13

In the case of anti-inflammatory IL-13, its serum concentration is increased in SZ subjects [54,97]; however, the SZ subjects in this study were treated with a cocktail of different antipsychotics (olanzapine, quetiapine, aripiprazole, fluphenazine, clozapine, chlorprothixene, amisulpride, ziprasidone, and haloperidol) [54]. A relevant point is that the subjects were treated with antipsychotics before and during the trial, which raises the question of whether the increase in anti-inflammatory cytokines is related to the use of antipsychotic treatment because the study itself does not clarify this situation. Also, high levels of IL-13 have been detected, particularly in patients with multiple episodes of SZ, in contrast to FES subjects and healthy controls. Differences (patients with multiple episodes of SZ in contrast to FES subjects and healthy controls) were maintained even after treatment with antipsychotics [98]. Thus, such differences could be part of the counter-regulatory response to the imbalance caused in SZ. In addition to the psychiatric disorders that come with SZ, their health is also poor in other aspects, such as poor nutrition and a sedentary lifestyle, among others [99], which raises the risk of comorbidities, such as cardiovascular disease (CVD) that increases their mortality rate [100]. A recent study determines the possible relationship between blood profile, biochemical factors, immune system imbalance, CVD factors and psychosis in FES subjects. One of the cytokines that increased in serum compared to healthy controls was IL-13, and the increase was correlated as a possible predictive prognostic indicator of SZ progression [101].

**Figure 1:**
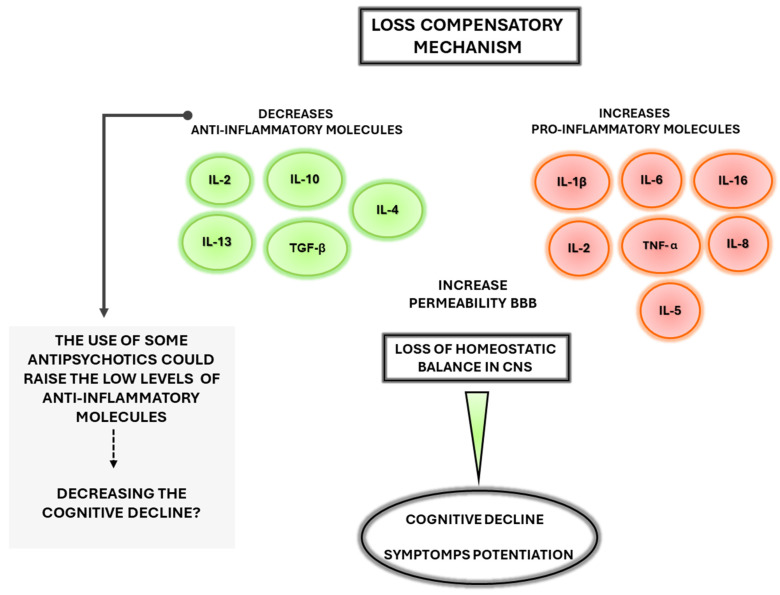
Representation of inflammatory imbalance and its contribution to cognitive deterioration in schizophrenia.

Studies on cytokine relationships with schizophrenia are summarised in Table 1 and Figure 1 shows the effects of imbalance inflammation in SZ.

## 3. Microglia and Antipsychotics: Their Relationship with Inflammation in Schizophrenia

### 3.1. Microglial Activation

Microglia are brain-resident macrophages and constitute the first step of cellular defence in the CNS [102]. The microglia function is also involved in synapse establishment during early development, and the number of neuronal cells regulation through synaptic pruning [103], also contributing to the survival of corticospinal and callosal neurons [104]. Microglia interact with neurons and vascular endothelial cells and contribute to migration cells and the developed cerebral wall [105]. Although the microglia are not traditionally part of the BBB, microglia communicate with cerebral vascular endothelial cells [106,107].

**Figure 2:**
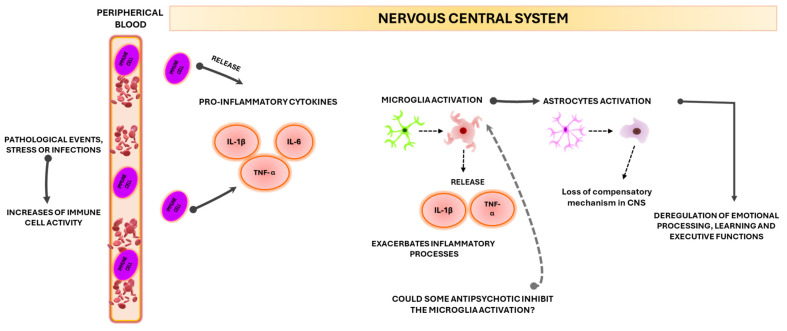
Representation of possible events that could cause microglia and astrocyte activation and contribute to the cognitive alterations in schizophrenia.

Under pathological events or infections, immune cells such as monocytes are recruited from the peripherical blood into the brain, which release inflammatory molecules with potential injuries to microglia and brain parenchyma (Figure 2). The exposure to inflammatory molecules IL-1β, TNF-α, and IL-6 induces morphologic changes in the microglia processes called microglia activation [108]; in this activated state, the microglia also release high levels of IL-1β and TNF-α [109]. Studies have found that peripherical inflammation increases IL-6 levels in the brain, which could cause a decrease in the firing of striatal medium spiny neurons, a component of the basal ganglia loop that has inputs from different regions of the cerebral cortex [110], participating in the emotional processing, learning and executive functions [111], areas related to psychiatric disorders. Microglia activation has been found in psychiatric disorders such as major depression, bipolar disorder, [112] and anxiety [113]. In the SZ context, more than a decade ago, Monji et al., proposed the microglia hypothesis, where stressful events induce microglia activation, increase pro-inflammatory molecules and release free radicals into the CNS, contributing to the pathophysiology of SZ [114]. In this vein, studies in animal models, have shown an increase in pro-inflammatory molecules due to the over-activation of microglia during foetal development [115,116,117] and adulthood. A prolonged inflammatory state by microglia activation during neurodevelopment may cause over-pruning of synapses. On the other hand, Ishizuka et al., [118] have found that the allelic variant Ala55Thr of CX3C chemokine receptor (CX3CR1), mainly expressed by microglia in the brain, have a significant association with SZ. CX3CR1 has a CX3C ligand, synthesised by neurons in the CNS, allowing crosstalk between microglia and neurons [119]. The CX3CR1-CX3C signalling dysfunction could interfere with critical events during development such as synaptic pruning [120]. A study has shown that CX3CR1 absence or deficiency in the hippocampus could be involved in cognitive dysfunction [121,122]. Another study carried out in twins where one twin was diagnosed with SZ showed a dysregulation in the gene expression profile of microglia that involved inflammatory molecules and upregulation of MHC class II. The MHC class II can attract lymphocyte T to brain regions where there is inflammation [123]; thus, an over-expression of MHC class II induces an increased lymphocyte infiltration in the brain, this can be supported by postmortem brain studies from SZ subjects where increased densities of lymphocytes T and B have been found in the cortex and subcortical white matter [124]. Also, a neuroimaging study has reported neutrophil infiltration in FEP subjects and a possible connection with reduced grey matter and enlarged ventricles [125]. In addition to an inflammatory event generated by immune cell infiltration in the brain, research suggests that infiltrating regulatory T lymphocytes (Tregs) could activate astrocytes to increase TGFβ secretion and promote an anti-inflammatory state, which could stimulate microglia to perform synaptic pruning because this activity is dependent of astrocyte-derived TGFβ [126]. This evidence supports the excessive synaptic pruning hypothesis that is time windows specific to neurodevelopment, where the loss of synapses in the cortex contributes to negative and cognitive symptoms and, additionally, to the disinhibition of projections in the mesostriatal area inducing an increase in dopaminergic activity and psychosis [127]. This evidence raises the question of whether synaptic pruning aberration in SZ only occurs during peri adolescence/adulthood and the prodromal stage, as was proposed four decades ago by Feinberg [128], or also occurs in SZ subjects lifelong in areas such as the olfactory bulb and hippocampus where there is synaptic pruning of adult-born neurons [129]. Thus, a permanent aberration of this process could contribute to increasing the cognitive deterioration in SZ due to the role of the hippocampus in memory function.

Another event that could lead to microglia activation is stress. The research in this area also comes from animal studies where the stressors exposition induces fast microglia activation (1 h acute stress exposition) in regions such as the thalamus, hypothalamus, hippocampus, and substantia nigra [130]. In the context of psychiatric disorder, the exposure to stress at an early age predisposes to the development of psychiatric disorders or behavioural problems in adulthood. In addition, subjects with psychiatric disorders are more susceptible to psychosocial stress. One longitudinal study has shown that psychosocial stress at an early age (9 years) is related to high levels of IL-6 and C reactive protein in serum, which was considered a risk factor for the development of psychiatric disorders such as depression and psychotic events in adulthood (18 years) [131]. SZ is a stress-vulnerable disorder, where psychosocial stress can be a risk factor for developing SZ when there is a genetic predisposition [132]. The psychosocial stress during specific time windows as adolescence could damage regions such as the prelimbic prefrontal cortex, an area involved in stress control, the damage in this area could be due to increasing dopaminergic activity engaged in the reward-related behavioural [133], which is altered in SZ and could contribute to negative symptoms. Xu et al. showed that the first episode of SZ subjects has a higher score in the perceived stress scale (PSS) and low levels of colony-stimulating factor 1 receptor (CSF1R) involved in the formation of microglia, thus stress could decrease the availability of microglia altering homeostasis in CNS, allowing peripherical inflammatory molecules into CNS and induce the microglia activation like a vicious circle. In the context of psychosocial stress, studies have shown dysregulation in the hypothalamic-pituitary-adrenal axis (HPA) in SZ subjects, increasing cortisol levels which potentially could generate microglia activation [134]. However, there are no studies that link microglia activation to psychosocial stress in SZ subjects.

On the other hand, microglia activation also can induce astrocytes to enter a reactive state (reactive astrocytes). Astrocytes are cells that participate in several functions in CNS, such as cellular communication among neurons, cellular metabolism, and regulation of neurotransmitter homeostasis, such as glutamate, are part of BBB [135]. The astrocytes are in a standby state in homeostatic conditions; however, when microglia are damaged, the astrocytes can substitute the microglia function through a compensatory mechanism, where the reactive astrocytes release proinflammatory molecules and may acquire phagocytic activity to engulf damaged microglia [136]. Also, a study showed that reactive astrocytes could act on microglia to induce the change from acute to chronic inflammation through astrocyte-derived secreted frizzled-related protein 1 (SFRP1), promoting the microglia activation and neuroinflammatory molecules release [137]. On the other hand, cortical microglia can modulate the function of astrocytes to a neuroprotective phenotype decreasing P2Y1 purinergic receptor expression. Further, exosomes containing microRNAs (miR-873a-5p) from reactive astrocytes [138] have an inhibitory effect on nuclear factor-κB (NF-κB) signalling in microglia, decreasing neuroinflammation. This effect could be a compensatory mechanism for the inflammatory events in the CNS.

In the context of SZ, an increase in NF-κB signalling has been observed [139]; thus, this compensatory mechanism of reactive astrocytes on microglia would not be present in SZ. But the results are controversial, for example, postmortem brains showed an increase in neuroinflammation in different cerebral areas but did not show a particular protein expression profile of reactive astroglia, the Glial fibrillary acidic protein (GFAP), produced after CNS injuries [140]. However, studies from Barley et al., [141] have shown high levels of GFAP expression in the mediodorsal nucleus of the thalamus, anteroventral, internal capsule, and putamen. Kim et al., [142] using monoamine oxidase B (MAO-B)–binding fluorine 18–labelled THK5351 show in vivo imaging of an increase in reactive astrocytes in the anterior cingulate cortex and left hippocampus from SZ subjects. Some studies postulate that astrocyte increase or decrease could be dependent on the brain area studied in SZ; however, whether psychosocial stress could induce an increase of microglia or astrocyte activation in SZ should be explored.

### 3.2. Antipsychotics and Microglial Activation

A relevant point that may modify inflammatory events in psychiatric disorders is the use of antipsychotics. Some antipsychotics act to inhibit microglia activation. Studies have shown that risperidone and haloperidol can suppress the release of inflammatory cytokines, such as IL-6 and TNF-α from activated microglia [143]. However, the results concerning haloperidol are contradictory, studies have also shown that haloperidol increases free radicals and decreases antioxidant enzymes in the brain, and molecules related to neuronal survival, such as brain-derived neurotrophic factor (BDNF) [144,145]. It has been documented that haloperidol treatment can induce or be involved in part in memory deterioration, thus we might think that memory damage could also be a consequence of activating microglia.

Aripiprazole can inhibit the increase of Ca^2+^ intracellular in the microglia due to exposure to IFNγ, a pro-inflammatory cytokine. The levels of elevated Ca^++^ intracellular are involved in microglia activation process, such as the release of cytokines, migration or microglia morphologic changes [146]. Chlorpromazine has been related to the voltage-gated proton channels (Hv1), which could cause current H^+^ inhibition, an event involved in the antioxidant enzyme’s function reducing the oxidants stage [147], thus low activity in these enzymes could potentially cause brain damage. Conversely, research on the second generation of antipsychotics has shown a prominent anti-inflammatory activity [148]. Thus, the effect of antipsychotics cannot be left aside when studying inflammatory processes in psychiatric disorders.

## 4. Brain Blood Barrier Dysfunction and Neurocognitive Impairment in Schizophrenia

Cognitive impairment in SZ subjects is present in several processes, such as verbal fluency, learning sequence problems and executive function. However, its mechanistic bases have not been completely elucidated. In this sense, the BBB can provide a possible explanation for the cognitive decline.

The brain has a microenvironment formed for barriers that allow maintenance of its functions and homeostasis in the CNS. These barriers are the brain–blood barrier (BBB), blood-cerebrospinal fluid (BCB) and arachnoid barrier [149]. The BBB is a selective and semi-permeable membrane formed by a neurovascular unit (NVU) composed of endothelial cells, pericytes, microglia, capillary basement membrane, and astrocytes end feet that separate the brain from the blood [150]. The endothelial cells are fastened by adherents and tight junctions (occluding junctions or zonulae occludes) and have no fenestrations. In homeostasis, the BBB impedes the influx of the molecules from peripheral blood into the brain. However, some organs in CNS have no BBB, they are named circumventricular organs [151].

### 4.1. BBB Disruption Measurement in SZ Patients

It has been reported that in psychiatric disorders, the BBB is altered, which could potentially cause parenchymal cerebral damage [152]. Studies carried out by Goldwaser et al., [153] in postmortem brains from SZ subjects show BBB disruption in the hippocampus and temporal cortex through IgG detection, the IgG extravasation from blood vessels to the parenchyma cerebral is used to determine the BBB permeability. Another methodology used for the BBB disruption study is the CSF/serum albumin quotient (Qalb). In another study, Campana et al., [154] show that 17,1% of 222 subjects with FEP have a high quotient Qalb, indicating a possible BBB alteration in this phase. In this context, one would wonder if the increase in permeability of BBB may be the beginning of cognitive decline in SZ. Still, more studies are necessary to determine if the quotient Qalb is a predictor for BBB dysfunction.

### 4.2. Protein mMarkers of BBB Disruption in SZ Patients

#### 4.2.1. S100β

The presence of S100β (Calcium binding protein, and neurotrophic cytokine secreted by astrocytes, microglia and neurons) in serum from SZ subjects has also been related to BBB disruption. In this regard, several studies show significant increases in peripheral S100β during disease development. For example, children and adolescents diagnosed with FEP, show such increases in S100β, in addition to an immune imbalance reflected in increased pro- and anti-inflammatory cytokines compared to healthy controls [155]. Recently, in adults who were hospitalised for an acute psychotic relapse, the dynamics of this protein have been determined in the serum of those SZ subjects at different times, where significant increases in S100β could be observed in admitted subjects. Then, levels decreased between admission and discharge but were still high compared to healthy controls. Finally, S100β decreased to normal levels three months after discharge [156], where the authors of this latest work conclude that it could be used as a tracer of how the disease evolves. Similar increases have been reported by Hong et al., [157], where their results showed high levels of S100β in serum SZ (n = 41) in comparison to healthy controls (n = 33); the authors conclude that increased levels of S100β could induce apoptosis due to the dual S100β function, which is supported by Li et al., [158]. These authors found that high concentration S100β (>100 ng/mL) cause the increases of IL-6 which could cause a chain reaction of neurodegenerative events.

This evidence exposes the effect that S100β could have on biological processes in central and peripherical nervous systems. However, more studies are necessary for a comprehensive understanding of S100β on psychiatric disorders and if S100β is a promising biomarker peripherical of BBB disruption due to the adipocyte also secreting S100β-mediated cold stress contributing to sympathetic system activation [159].

#### 4.2.2. Claudins

Studies on psychiatric disorders focused on the proteins of NVU, such as claudins (CLDN), have demonstrated that exposure to psychosocial stress is an event that induces immune activation, which increases the inflammatory molecules causing incorrect expression of CLDN5 in endothelial–endothelial junctions in the NA [160]. This brain region is related to the reward system and is involved in the cognition processing motivation [161]. The motivation deficit is one of the negative symptoms in SZ. Thus, the question arises if the BBB disruption could be partly responsible for the impairment of cognition processing motivation in SZ subjects. Cytokines such as TNF-α and IFN-γ can impact the expression of occludin [162].

The role of CLDN5 as a marker of BBB disruption in SZ has also been discussed. The SNP rs10314, located in the 3′-UTR region of the *Claudin 5* gene, would have an incidence in the development of SZ [163,164]. This SNP has recently been found to decrease CLDN5 expression by 75% in vitro [165]. In fact, 30% of subjects with 22q11 deletion syndrome (22q11DS) have SZ, and one of the genes that is lost is the *CLDN5* gene (22q11.21 region) [165]. Also, studies on mice designed to suppress Cldn5 expression show that in hippocampal/prefrontal cortex knock-in and inducible knockdown mice, CLDN5 mRNA and protein levels decreased significantly. Whereas in knock-in mice, hippocampal and prefrontal cortex extravasation increased, i.e., BBB disruption occurred. Knockdown mice even displayed psychosis-like behaviour [166]. Thus, the deletion of Cldn5 in mice shows the importance of tight junctions in BBB integrity and proper functioning of the CNS. Interestingly, Greene et al., [166] observed that antipsychotic drugs increased Cldn5 expression in a dose-dependent manner in vitro, an effect that would be mediated by the Wnt pathway, a pathway responsible for generating and maintaining the BBB [167], because the presence of relevant pathway proteins, such as Axin-2 [168] and Sox-17 [169], which could be due to the low transcription and translation of CLDN5, compromising BBB function, with less CLDN5 being released under these conditions.

#### 4.2.3. GFAP

Also, GFAP has been related to the disruption of BBB, in this sense, a systematic study carried out in postmortem brain from SZ showed an increase in neuroinflammation in different cerebral areas and cell types such as astroglia where GFAP, a marker of astroglial cell activation, produced after CNS injuries [170] does not show a particular protein profile expression in SZ [140]. However, strategies such as subclassification of study subject groups could lead to changes in the results, as Catt et al., 2014 [171] initially, no significant differences in GFAP were observed between healthy controls and SZ subjects. However, when selecting groups of SZ subjects with and without markers of neuroinflammation in addition to astrocyte morphology, significant differences in GFAP were observed in SZ subjects with markers of neuroinflammation and hypertrophic astrocytes compared to SZ subjects without neuroinflammation [171]. A similar situation is observed in the detection of a GFAP expression pattern in fluids, where it has been reported that there are no significant differences in both serum and CSF of SZ subjects compared to healthy controls [172]. On the other hand, significant decreases in serum GFAP in SZ subjects compared to healthy controls have also been reported, both in studies with a small Turkish cohort [173] and with a much larger Chinese cohort [174]. Therefore, there are also inconsistencies in the observations of GFAP at the fluids in SZ. Thus, the GFAP expression in SZ could be discussed, and its increase or decrease could be dependent on the brain area studied in SZ and the presence of GFAP at the fluid level due to BBB disruption should also be included, where more clinical trials are needed to find a consensus on this point.

### 4.3. Hypothesis for BBB Disruption

#### 4.3.1. BBB Disruption and Inflammation

Further, it has been reported that the complex IL-6 and its receptor can cross the BBB, eventually increasing the permeability and inducing inflammatory events in the CNS. In SZ subjects, high levels of IL-6 have been reported and could be implicated in the BBB disruption. On the other side, the molecular mechanism of S100β for increasing the IL-6 expression could be mediated by neuronal NF-κB, an inducible transcription factor where the canonical pathway is related to inflammatory processes, furthermore, it can induce the pro-inflammatory genes and inflammasome activation [175]. In this line, a study by Gober et al., [176] showed increased inflammasome proteins in microglia and Liu et al., [177] showed a correlation among that nod-like receptor pyrin domain-containing protein 3 (NLRP3), an inflammasome protein that mediates pro-inflammatory cytokines, and cognitive impairment. Moreover, high levels of S100β can induce increased activity of nitric oxide (NO) and nitric oxidase synthase (iNO). In this context, if astrocytes are exposed to a high concentration of iNO it can lead to enhanced NMDA receptor-mediated neurotoxicity, a hypothesis that underlies SZ. Research carried out in neurodegenerative diseases also found that high levels of S100β can potentially generate gliosis mediated by NO production from astrocytes and microglia. Interestingly, the S100β effects also could affect the peripheral system, inducing damage in several tissues, in addition, S100β can cause oxidative stress [178] and apoptosis [179].

#### 4.3.2. BBB Disruption and Gut Dysbiosis

**Figure 3:**
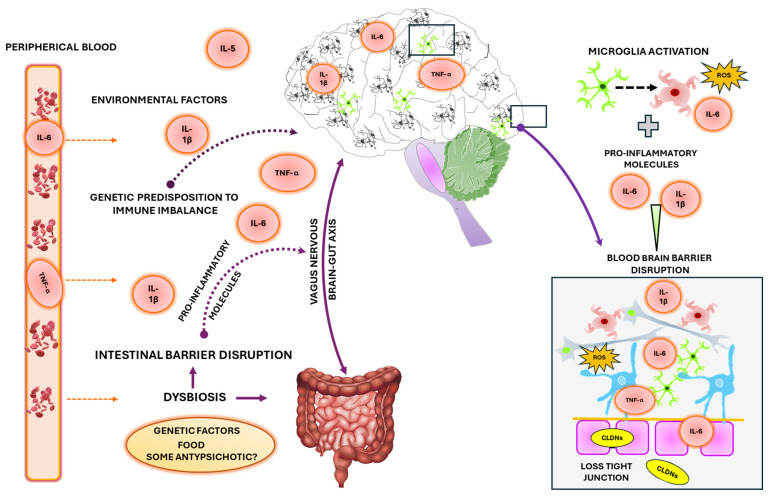
General representation of possible events that could induce the BBB disruption and contribute to the cognitive decline in schizophrenia.

Last, numerous theories have been raised about the dysregulation in the gut microbiota and its effect on the disruption in the intestinal and BBB. The dysbiosis in the microbiome has been reported in SZ, and studies have found an increment in gastrointestinal Inflammation, which potentially could damage the intestinal barrier [180]. The intestinal inflammatory molecules produced by dysbiosis microbiota could influence the CNS due to the gut-brain axis impacting the BBB structure and brain parenchyma (Figure 3). A point to consider is the use of antipsychotics [181] due to their influence on gut-bacterial which could eventually contribute to cognitive damage in SZ [182]. Furthermore, the gut microbiota can be modulated by stress and precisely SZ subjects are susceptible to psychosocial stress. However, more studies in this area are necessary to evaluate whether deregulation in the components of microbiota can also be responsible for the symptomatology of SZ.

## 5. Genetic Alterations in Schizophrenia

As we have documented in the previous sections of this review, the contribution of genetic alterations to the development of schizophrenia cannot be omitted. The GWAS study found 108 loci altered in this disorder [33]. Furthermore, in SZ patients, mutations and polymorphisms have been found in genes related to myelination [183], neurotransmitter receptors [184], ferroptosis [185] and immune response (Table 2). Interestingly, changes in methylation of immune-related genes have also been detected in a subpopulation of FEP patients [186]. In contrast, differential expression for a set of immune-related genes has been found in postmortem brain tissue of SZ patients [187,188], human IPS-derived neural progenitors and neurons from SZ patients and whole blood of early-onset SZ patients [189] (Table 2).

## 6. Anti-Inflammatory Treatment, Could Be the Solution?

As previously mentioned, there is growing evidence that inflammatory events and their imbalance are relevant factors, and their effects on CNS could contribute to elucidating, in part, the aetiology of SZ. For example, it has been determined that IL-1 β [45], IL-2 [192], IL-6 [193] or its mRNA [194], and IL-8 [195] could be candidates to be used as possible markers for SZ. In fact, modulation of peripheral inflammation or neuroinflammation has been proposed as a potential treatment for neuropsychiatric pathologies such as depression or SZ [196]. Focusing mainly on the control of these pro-inflammatory molecules, different strategies have been proposed, such as the use of nonsteroidal anti-inflammatory drugs (NSAIDs), such as Celecoxib [197,198,199], the N-acetylcysteine (NAC) [200,201], or the classic Acetylsalicylic acid (Aspirin); however, the latter did not have good results [202,203]. Antibiotics with anti-inflammatory effects, such as Minocycline, have also been proposed to treat SZ [204], with mixed results, this depends on whether it is used as an adjuvant [205,206] or as a stand-alone treatment [207,208]. Because of their specificity, the most sophisticated strategies use monoclonal antibodies to block the effect of specific cytokines, such as Adalimumab [209], an antibody against TNF-**α** that blocks its signalling pathway. Canakinumab, antibody against IL-1β, interfering with its bioavailability [210] or the antibody Tocilizumab, which blocks the IL-6 receptor [211], however, the latter does not seem to show relevant effects on SZ. 

Recently, worthwhile results have been reported using a specific inhibitor of the TGF-β1 receptor, RepSox, a molecule capable of down-regulate inflammatory genes and up-regulate BBB genes such as Cldn5 in vitro [212] this molecule can up-regulate Cldn5 at the mRNA and protein level and was able to stabilise the BBB in rodents with disrupted BBB [213]. These results are promising, so a possible use of RepSox could be tested in future human trials and treatments to restore BBB functionality in subjects with SZ.

## 7. Conclusions or Future Considerations

In psychiatric disorders, several studies support the association between inflammation and BBB disruption, the deregulation in these events could impact the parenchymal cerebral contributing to pathways or biological process dysfunction involved in cognitive processes such as verbal fluency, learning sequence problems and alteration in executive functions (concentration, prioritisation). SZ is known to be an “inflamed brain”, in this sense, the evidence exposed in this review supports the influence that inflammatory molecules may have on the aetiology or progression of SZ and its relationship with cognitive decline.

Our findings are also summarised in Table 1, where we present the changes in cytokine levels and their possible relationship with positive and negative symptoms in schizophrenia. Furthermore, we raise the question of whether the loss of compensatory mechanism in the anti-inflammatory response could be a relationship with cognitive decline in schizophrenia (Figure 1). On the other hand, factors such as stress and injuries can activate microglia, increasing the levels of several cytokines with a possible role in astrocyte’s activation and the loss of the compensatory mechanism, generating a chain reaction among activation of microglia and astrocytes (Figure 2). Genetic alterations (Table 2) could induce damage to the brain parenchyma, and BBB disruption and contribute to the occurrence of different symptoms and cognitive deterioration in SZ (Figure 3).

Also, the study of the antipsychotic effects on intestinal microbiota in SZ subjects is a point to consider due to its role in dysbiosis, a biological event that increases the release of inflammatory molecules to the CNS and potentially may generate the BBB disruption (Figure 3).

From this review, the question arises of whether there is a possibility of anti-inflammatory treatment in a specific phase of SZ such as in the prodromal phase or first psychotic event, being a highly inflammatory event targeted for future treatment. Moreover, is it possible to obtain inflammatory biomarkers to predict a first psychotic event?

## Figures and Tables

**Table 1:** Pro and anti-inflammatory cytokines found to be altered in SZ patients.

Molecule	PANSS Symptoms	Changes Observed	Type of Patient	Fluid/Tissue	Reference
TNF-α	Negative	↑	W/treatment	Serum	[36]
	Negative	↑/↓	FEP, W/treatment	Serum	[37]
IL-2	Negative	↑	W/treatment	Plasma Serum	[42]
IL-1β	Positive and Negative	↑/No change	FEP, W/treatment, Relapse	Serum	[45]
	Negative	No change/↑	FEP, UHR	Serum	[46]
IL-5	Not mentioned	↑	W/treatment	CSF, Serum	[54]
IL-6	Negative	↑	WO/treatment	Serum, plasma, CSF	[56]
	Not significant	↑	W/and WO/treatment	Serum	[57]
IL-8	Negative	↑	FES	CSF, Serum	[63]
IL-16	Not mentioned	↑	W/treatment	Serum	[69]
	Negative	↑	WO/treatment	Plasma	[45]
TGF-β	Not significant	↑/↓	FES, W/treatment	Plasma	[73]
	Negative	↑/↑/↓	FEP, Relapse, W/treatment	Serum	[70]
	Not significant	↓	Treatment resistant	Plasma	[76]
IL-10	Negative	↓	FES, WO/treatment	Serum	[77]
	Not significant	↑	WO/treatment	Plasma	[79]
IL-4	Negative	↑	FEP	Serum	[91]
	No correlation	↑	FEP, Relapse	Serum	[92]
	Not mentioned	↓/↓	FEP, Relapse, W/treatment	Serum	[93]
IL-13	Not mentioned	↑	W/treatment	Serum	[97]
	Not mentioned	↑	W/treatment	CSF, Serum	[54]
	Not significant	No change/↑	FES, Multi-episode	Serum	[98]
	Negative	↑	FES	Plasma	[101]

W/treatment: with treatment; WO/treatment: without treatment; UHR: ultra-high risk subjects. The up and down arrows indicates increases and decreases respectively.

**Table 2:** Immune-related gene changes associated with SZ.

Results	Target Genes	Methodology	Tissue	Reference
Polymorphisms associated with higher risk	***IL1A*** (rs1800587), ***IL1B*** (rs4848306), ***IL1RN***(rs4251961), ***IL2*** (rs2069762), ***IL6R*** (rs4537545), ***IL8*** (rs1126647), ***IL10***(rs1800871, rs1800872, rs1800896, rs6676671, rs1179263), ***IL33*** (rs1179263), ***IFNG*** (rs2069718), ***TGF1β*** (rs1800470)		Blood	[40,75,85,86,87,190]
	***TNFα*** (rs1800629)			[38]
	***IL1B*** (rs1143633)			[51,52]
Altered methylation	Non specified	Epigenome-wide profiling with a DNA methylation array	Blood	[186]
Differential expression	*GRIN1*, ***P2RX7***, ***CYBB***, *PTPN4*, *UBR4*, ***LTF***, *THBS1*, *PLXNB3*, *PLXNB1*, *PI15*, *RNF213*, *EIF4A2*, ***CXCL11***, ***IL7***, *ARHGAP10*, *TTR*, and ***TYROBP***	Bioinformatic and machine learning analysis	Brain	[187]
Differential expression	*S100A2*, ***CCL14***, ***IGHA1***, *BPIFA1*, *GDF15*, ***IL32***, *BPIFB2*, ***HLA-DRA***, ***S100A8***, ***PTX3***, *TPM2*, ***TNFRSF12A***, *GREM1*	Single cell transcriptomics	Brain	[188]
Differential expression	*FN1*, *COL1A1*, *COL3A1, COL1A2*, *COL5A1*, *COL2A1*, *COL6A2*, *COL6A3*, *MMP2*, *THBS1*, *DCN*, *LUM*, ***HLA-A***, ***HLA-C***, and *FBN1*	RNA-seq datasets bioinformatic analysis	hIPS-derived NPC	[191]
Differential expression	***CCL8***, *PSMD1*, *AVPR1B* and *SEMG1*	Microarray data from a whole-genome mRNA	Blood	[189]

Genes in bold are the ones verified with immunological functions.
